# Patient and caregiver experiences of living with acute hepatic porphyria in the UK: a mixed-methods study

**DOI:** 10.1186/s13023-021-01816-2

**Published:** 2021-04-26

**Authors:** Liz Gill, Sue Burrell, John Chamberlayne, Stephen Lombardelli, Jordanna Mora, Nicola Mason, Marieke Schurer, Madeline Merkel, Stephen Meninger, John J. Ko

**Affiliations:** 1 British Porphyria Association, Durham, UK; 2 Alnylam Pharmaceuticals, Alnylam UK Ltd, Braywick Gate, Maidenhead, SL6 1DA UK; 3 Alnylam Pharmaceuticals, Cambridge, MA USA; 4 BresMed Health Solutions Ltd, Steele City House, Sheffield, UK; 5 BresMed Netherlands B.V, HNK Utrecht CS, Utrecht, Netherlands

**Keywords:** Porphyria, Acute hepatic porphyria, Qualitative, Quantitative, Quality of life, Caregiver experience, Burden of illness, Pain, Chronic symptoms, Neuropathy

## Abstract

**Background:**

This study used quantitative and qualitative research methods to analyze how acute hepatic porphyria (AHP) affects patients with varying annualized porphyria attack rates. The overall impact of AHP on patients and caregivers, including their quality of life, was explored. The nature and treatment of acute attacks, experiences of long-term heme arginate treatment and access to other appropriate treatment, and the extent of and treatment for chronic symptoms were also investigated within this study.

**Methods:**

Patient and caregiver data were collected via an online survey of members of the British Porphyria Association, followed by an optional 1-h telephone interview.

**Results:**

Thirty-eight patients and 10 caregivers responded to the survey. Of those, 10 patients and three caregivers completed follow-up interviews. Overall, 19 patients (50%) had experienced an acute attack within the previous 2 years, and the severity and types of symptoms experienced during or between acute attacks varied considerably. There were no clear definitions among patients for ‘mild’ or ‘severe’ attacks. Treatments and treatment settings used to manage attacks also varied. Following unsatisfactory care experiences at hospitals, some patients reported avoiding further hospital services for later attacks. Therefore, using settings of care as a measure of attack severity should be avoided. Ninety-four percent of patients also experienced chronic symptoms, which were as varied as acute attacks. Pain was the predominant chronic symptom and was managed with opioids in severe cases. Regardless of AAR, porphyria heavily impacted the daily lives of patients and caregivers. Although patients experiencing frequent attacks generally endured a greater impact on their daily life, patients with less frequent attacks also experienced impacts on all domains (social, leisure activities, relationship with family, relationships, psychological wellbeing, finances, employment, and study). Caregivers were most affected in the finance, relationships with family, and employment domains, and just over half of the caregivers reported a moderate impact on their psychological wellbeing.

**Conclusions/implications:**

The burden of illness with AHP is high across all patients, regardless of frequency of attacks, and AHP negatively affects patients and caregivers alike.

**Supplementary Information:**

The online version contains supplementary material available at 10.1186/s13023-021-01816-2.

## Background

Porphyrias are a group of largely hereditary metabolic disorders caused by a defect in heme biosynthesis and are classified according to the principal site of expression, as either hepatic or erythropoietic [1]. Acute hepatic porphyria (AHP) is characterized by acute attacks of pain (usually abdominal), autonomic symptoms (e.g., hypertension, tachycardia, nausea, vomiting), and neurologic manifestations such as weakness, confusion, and seizures [2, 3]. AHP attacks are potentially life-threatening, and some patients also develop chronic, debilitating symptoms that negatively impact daily functioning and quality of life [3].

The prevalence of symptomatic AHP in the UK is estimated to be 1 in 100,000 [4]. However, lack of clinical recognition of AHP and the use of inappropriate diagnostic tests may result in underdiagnosis and underestimation of prevalence calculations [5, 6]. Of the four types of AHP, acute intermittent porphyria (AIP) is the most common and most associated with frequent acute attacks. Variegate porphyria (VP) and hereditary coproporphyria (HCP) present with cutaneous manifestations alongside the acute neurologic manifestations [7]. The fourth type, aminolevulinic acid dehydratase deficiency porphyria (ADP), is autosomal recessive, with only a handful of case reports in the literature [7].

The results presented here are based on the AHP treatment options available to the surveyed patients at the time of the study, at which time there were no approved treatments for the prevention of AHP attacks. Management of AHP was limited to environmental and symptom management, including avoidance of factors that trigger an attack, treatment of the acute attacks, management of pain and chronic symptoms, and protecting skin from the light in cutaneous manifestations (VP and HCP) [5, 8]. Treatment decisions were largely influenced by patient severity, most commonly defined by frequency and severity of attacks [9, 10]. As a result, previous studies have stratified patients by frequency of attacks, known as the annualized attack rate (AAR) and defined as annualizing the number of AHP attacks over a specified time period. For instance, patients may be stratified as those with an AAR of either ≥ 3 or > 4 attacks per year (‘recurrent attacks’) and those with fewer or no attacks [11–13]. Those with recurrent attacks represent approximately 3–5% of the overall patient population [9].

Several studies have characterized the disease and disease burden of patients with AHP [2, 3, 7, 11–14], with a focus on patients experiencing recurrent attacks [3, 11, 12]. Disease characteristics in patients having fewer attacks are less well understood [13]. This study used both quantitative and qualitative research methods to build on the existing research to analyze the effects of AHP on patients with different frequencies of AAR. The nature of acute attacks, chronic symptoms, and subsequent impacts of the burden of the condition on the patients’ and caregivers’ quality of life, physical functioning, work, social and family life, personal relationships, and perceptions of the healthcare system were considered as part of the analysis.

## Results

**Fig. 1:**
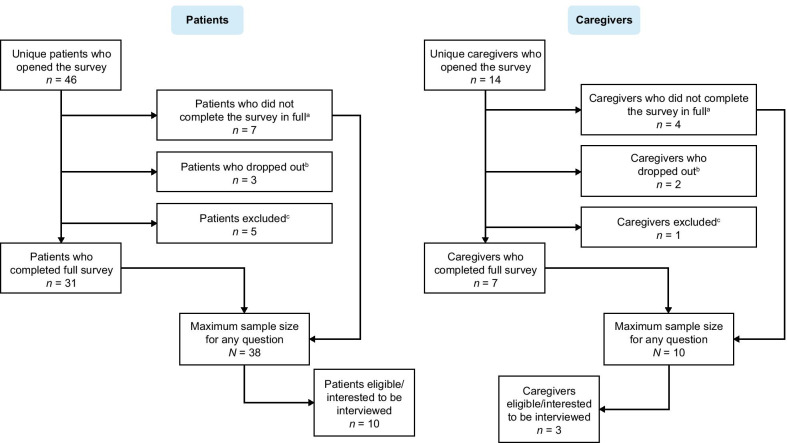
Patient and caregiver flow diagram. ^a^Where not all mandatory questions were completed. ^b^Where the survey was closed at one of the survey eligibility questions. ^c^Where patients/caregivers did not meet the eligibility criteria

A total of 38 patients and 10 caregivers responded to the survey. Of those, 10 patients and three caregivers completed the follow-up interviews. Figure 1 provides a full overview of the patient and caregiver samples. Most participants (both patients and caregivers) in the online survey and subsequent interviews were female (90% and 69%, respectively). Participants had or were caring for someone with AIP, VP, or HCP (survey: 77%, 21%, 2% and interviews: 92%, 8%, 0%, respectively). All interviewed patients had experienced at least one attack in the past 2 years that required hospitalization, an urgent healthcare visit, or treatment with intravenous heme arginate at home. Across 28 patients in the survey population, a mean of five attacks per patient was reported over the previous 2 years. Full patient and caregiver demographics and clinical characteristics for the online survey and follow-up interviews can be found in Tables 1 and 2, respectively. Where the interviews included patient and caregiver descriptions of the impact of porphyria, a breakdown of the frequency of the patients’ attacks is shown in Additional file 1: Table S1.

1. **Acute porphyria attacks**

**Key findings** The severity and types of symptoms during or between acute attacks varied considerably. Among patients, there was no clear definition reported for ‘mild’ or ‘severe’ acute attacks; definitions varied per patient and were dependent on individual disease experiences.

**Table 1:** Demographics and characteristics from the online survey

Participant characteristics	Patient (*n* = 38)	Caregiver (*n* = 10)
Sex, *n* (%)		
Male	2 (5)	3 (30)
Female	36 (95)	7 (70)
Type of porphyria		
AIP	28 (74)	9 (90)
VP	9 (23)	1 (10)
HCP	1 (3)	0 (0)
Mean age when experiencing first symptoms, years (range)^a^	22.4 (10–45)	N/A
Mean age when diagnosed, years (range)	23.7 (1–48)	N/A
Mean years of caring for patient, years (range)	N/A	13.3 (4–24)
Relationship to patient, *n* (%)		
Parent	N/A	6 (60)
Partner/spouse	N/A	3 (30)
Close friend	N/A	1 (10)
Frequency of attacks within the previous 12 months, *n* (%)		
AAR 0 or unsure	24 (63)	N/A
AAR < 3	9 (24)	N/A
AAR ≥ 3	5 (13)	N/A

Note: Data within the caregiver column are relevant to the patients with AHP for whom they are caring

^a^Due to the small patient population, the survey did not ask patients or caregivers to provide their current age for anonymity purposes

**Table 2:** Demographics and characteristics from the telephone interviews

Participant characteristics	Patient (*n* = 10)	Caregiver (*n* = 3)
Sex, *n* (%)		
Male	1 (10)	3 (100)
Female	9 (90)	0
Mean age, years (range)	37.3 (24–57)	40.3 (30–55)
Work status, *n* (%)		
Unemployed	3 (30)	N/A
Voluntary work	1 (10)	N/A
Student	1 (10)	N/A
Employed	5 (50)	N/A
Marital status, *n* (%)		
Single	3 (30)	N/A
Partnership	1 (10)	N/A
Married	6 (60)	N/A
Type of porphyria, *n* (%)		
AIP	9 (90)	3 (100)
VP	1 (10)	0
HCP	0	0
Mean age when experiencing first symptoms, years (range)	22.3 (14–33)	N/A
Mean age when diagnosed, years (range)	23.8 (13–33)	N/A
Relationship to patient, *n* (%)		
Parent	N/A	0
Partner/spouse	N/A	3 (100)
Close friend	N/A	0
Number of attacks in the last 2 years, *n* (%)^a^		
1–3	5 (50)	1 (33)
4–6	2 (20)	1 (33)
7–9	0	0
≥ 10	2 (20)	0
Unclear	1 (10)^b^	1 (33)
Recurrence of attacks within the previous 12 months, *n* (%)		
AAR < 3	4 (40)	2 (66)
AAR ≥ 3	6 (60)	1 (33)
Regular prophylactic treatment, *n* (%)		
No	5 (50)	2 (66)
Yes, heme arginate	4 (40)	1 (33)
Yes, GnRH agonist	1 (10)	0

Note: Data within the caregiver column are relevant to the patients with AHP for whom they were caring

^a^Caregiver-reported data were used where possible to complete the caregiver column; in one case this deviated from the patient-reported data. Whenever someone provided a range of attacks over a certain period, the average was used (e.g. ‘around once every four months’ was interpreted as three times a year)

^b^Patient E (≥ 3 attacks per year) experienced six to 10 ‘bad ones’ per year since being diagnosed but had a good year in 2016–2017. Therefore, it is unclear how many attacks were experienced in the last 2 years

Among the online-survey group (*n* = 38), 84% (*n* = 32) reported experiencing an acute attack since being diagnosed with AHP, 5% (*n* = 2) reported never experiencing an attack, and 11% (*n* = 4) were unsure if they had ever experienced an acute attack. Of those patients who had experienced an attack since diagnosis, 81% (*n* = 26) reported experiencing an attack within the past 2 years.

In the interviews, patients were asked to further define the characteristics of an ‘acute attack’ by describing the symptoms they tended to experience. Table 3 summarizes these symptoms. Specifically, the symptom of intense pain during attacks was experienced and detailed by patients suffering from varying frequencies of annualized porphyria attack rates (AARs) (Table 4).

**Table 3:** Summary of patient-reported description of attack symptoms during the interviews

Type of symptoms	Patients mentioning symptoms (*n* = 10) (%)	Patient-reported symptom descriptions
Pain	100	Abdominal pain Stomach pain Headaches
Psychiatric symptoms/ mental status changes	70	Confusion and memory problems Psychotic episodes (hallucinations and self-harm) Paranoia
Digestive system	70	Nausea and vomiting Bowel symptoms Diarrhea and constipation Loss of appetite
Paralysis and muscle weakness	60	Onset of long-term paralysis Muscle weakness Loss of sensation in lower limbs
Other symptoms	70	Collapsing (including ‘fits’, ‘passing out’, and ‘seizures’) and dizziness ‘Flu-like’ symptoms, such as joint stiffness, joint pain, coughing, and sweating Insomnia^a^ Dark urine Rashes

Note: ^a^It was unclear according to the patient if this was a symptom caused by an acute attack or by another cause

**Table 4:** Quotes relating to acute pain as an experienced symptom

Quote (identifier)
“A [labour] contraction that doesn’t end […] I can kind of handle pain, but this was just off the scale […] it was like blinding pain […] feels like someone’s just tearing your insides from within all the time, constantly […] like burning shears.” (Patient I)
“Just constant stabbing, whipping, burning pain across my ribs and my abdomen […] then spread up to my lungs. So, it feels like I can’t breathe properly. Spreads down into my lower abdomen, my legs. And the intensity of the pain is anywhere from eight to ten out of ten […] it would feel like someone was pouring acid on my intestines and then ripping them open. And then around my ribs and my lungs particularly, it would feel like someone was scraping my ribs with knives. And then in my spine I would feel like a hot poker, pressing into my spinal cord and sending shooting pains up and down my body.” (Patient E)

Questions relating to the severity of acute attacks were not included in the survey. In the interviews, patients stated that the severity of acute attacks can vary, and it became clear that there is no consensus definition for what constitutes a ‘mild’ or ‘severe’ attack. Rather, the severity of attack was based on patients’ unique, subjective disease experience. One consideration of the severity of an acute attack identified through the interviews was based on whether the acute attack could be managed at home or if hospital admission was required (caregiver, *n* = 1; patient, *n* = 5) (Table 5). Others considered attack severity by the type of symptoms they experienced (patients, *n* = 4) or the duration of the attack (patient, *n* = 1) (Table 5).

2. **Treatment of attacks**

**Key findings** Treatment to manage attacks varies, and previous hospital experiences can affect whether the patient chooses to return to hospital or tries to manage attacks at home. There is considerable variability in satisfaction with access to treatment. Patients expressed gratitude at being able to receive treatment with heme arginate; however, long-term heme arginate was not without practical and physical impact.

**Table 5:** Quotes relating to defining the severity of acute attacks

Quote (identifier)
“Severity is variable. So, [my wife] had multiple attacks in a single year and […] she’s been diagnosed with porphyria since 2008. So, we’ve had 100 of these things. I tend to group them into ones that have required hospitalisation and ones that haven’t required hospitalisation […] Ones that have required hospitalisation – most of the time […] were sort of […] what I would call, ‘the massive attacks’ or ‘the mega attacks’. [My wife] has had lots of ‘little’ attacks, that haven’t required hospitalisation, where she’s just had lots of pain and, sometimes with the pain she’d also get sickness.” (Caregiver C)
“For myself it would be amount of symptoms that I’m having at one given time. So, if I’m having abdominal pain, diarrhoea, migraine, pain in my limbs, shooting pains, kind of insomnia that would be […] quite an average one for me. If I’m then starting to get into psychiatric territory and migraines and collapsing, with all of those other symptoms […] you now have to go to the hospital, get someone involved.” (Patient D)
“If it is a very minor attack, which they kind of tend to be now, my doctors have told me “if you feel you can manage it at home, do so” – this sounds really bad, I know, but a lot of the people I know with porphyria don’t want to go to A&E every single time because it just feels like a battle with the staff at A&E. If it then becomes so bad that I cannot function, then yeah I need to go to hospital.” (Patient D)

**Fig. 2:**
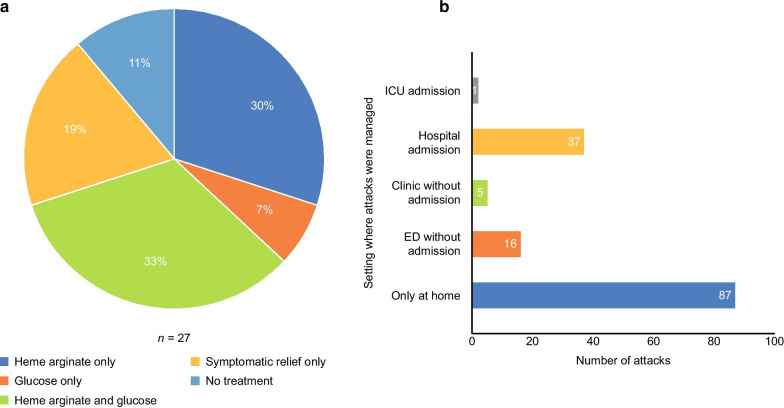
Management of acute attacks. **a** Treatment to manage acute attacks;** b** setting in which acute attacks were managed^a,b,c^. ^a^Patients were able to select more than one setting in which their attack was managed. ^b^Twenty-three patients responded to this question, reporting a total of 146 attacks. ^c^The survey did not differentiate between symptomatic treatment or prescribed treatment at home with heme arginate

The majority of patients in the survey (84%, *n* = 27/32) were able to specify the treatments required to manage their acute attacks (Fig. 2a), and of these, 71% of patients (*n* = 23) were able to specify the setting in which their acute attacks were treated (Fig. 2b). The majority of these attacks (60%, *n* = 87) were managed at home.

The interviews provided examples of when patients felt their acute attacks could no longer be managed at home, including when the patient could no longer eat or increase their carbohydrate intake any further (caregiver, *n* = 1; patient, *n* = 3), or when the pain could no longer be managed at home (caregiver, *n* = 1; patient, *n* = 1) (Table 6). Three patients and one caregiver noted that while opioid medication was available at home, it was not considered effective for managing the pain associated with a severe attack.

**Table 6:** Quotes relating to management of acute attacks

Quote (identifier)
“Once I start vomiting, I would go to the hospital, because […] if I couldn’t get any food into me, it was a bit of a lost cause and I would have to get treatment straight away.” (Patient E)
“[…] I try and take the paracetamol in the house and that doesn’t work. I do everything I can but when it gets so bad it quickly happens […] within 30 minutes I can be [in] non-stop pain dialling for an ambulance.” (Patient B)
“The pain was excruciating, the pain killers, intramuscular morphine […] that didn’t touch it!” (Patient G)
“I know that no common painkillers will take [the pain] away […]” (Patient J)

**Fig. 3:**
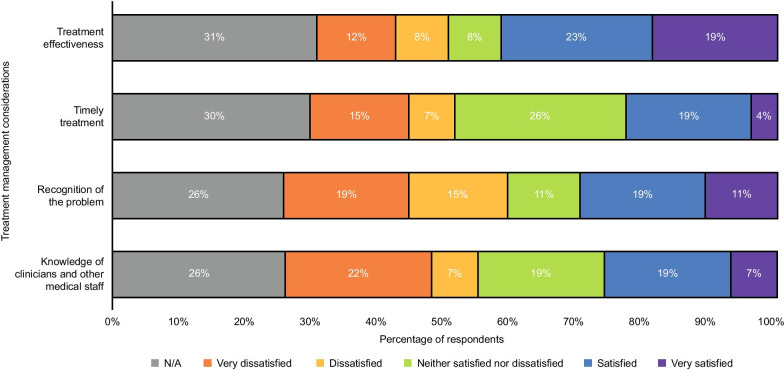
Patient-reported satisfaction with the management of their acute attacks within the past 2 years (n = 27)

Figure 3 displays the results of the patients (*n* = 27) from the survey who reported their satisfaction in relation to how their acute attacks were managed by treatment over the past 2 years. Combined values of patients who were ‘dissatisfied’ and ‘very dissatisfied’ with the effectiveness of treatment for their acute attacks (43%) were broadly similar to the combined values of patients who were ‘satisfied’ or ‘very satisfied’ (42%). Two patients and one caregiver stated dissatisfaction in treatment management was due to a lack of understanding and recognition of AHP in Accident & Emergency (A&E), which resulted in delays to appropriate treatment (Table 7).

**Table 7:** Quotes relating to the challenges in accessing appropriate treatment

Quote (identifier)
“A lot of the people I know with porphyria don’t want to go to A&E every single time because it just feels like a battle with the staff […]” (Patient D)
“[When stating that the patient’s pain relief is morphine through an IV drip] they look at you like you are some kind of drug addict […] at the end of the day I’ve gone to the hospital for a reason and that reason is to control my pain and get better, so that I can go home. But I think they just don’t – there is no understanding of it.” (Patient H)
“Right at the beginning, when we went into hospital, we didn’t have a letter from [our doctor] explaining [that my wife had AHP], because it was very difficult to turn up at an A&E and go ‘by the way, my wife has got porphyria, and this is really painful’ […] We slowly learnt, first of all, not to go to our local hospital, but to drive a little bit further to [the specialist centre], which is where [our doctor] is based.” (Caregiver C)

Limited questions relating to prophylactic heme arginate use were included within the survey. However, four of the 10 patients included within the interview phase received prophylactic heme arginate treatment (all had ≥ 3 attacks per year); three patients received it every other week, and one patient had a weekly regimen. Treatment infusions were administered at home (*n* = 3) through either self-administration, nurse administration, or caregiver administration. The fourth patient was required to go to hospital for their infusion due to difficulties in accessing the central venous line at home. The patients described the difficulties associated with repetitive heme arginate use, including drug administration and iron accumulation, in Table 8.

3. **Chronic symptoms**

**Key findings** Chronic symptoms were common in most patients, regardless of AAR, and were as varied as acute attacks. The use of analgesics was the most frequently reported method to manage chronic pain associated with AHP. Patients reported a lack of treatment options to manage their other symptoms, including fatigue, tiredness, lack of concentration, and confusion.

**Table 8:** Quotes relating to the challenges of long-term prophylactic heme arginate (off-label) treatment

Quote (identifier)
“I am unable to work full-time because I have to have at least one day off for my hospital haem arginate and the maintenance. It’s very, very difficult to plan anything long term, like […] holidays, family holidays, wedding attendance […] [travelling to meet with the consultant] is very tiring in itself and costly. Financially, it’s put a strain, obviously going from a full-time position to part-time.” (Patient B)
“Yes, I still do [the haem arginate infusions] every two weeks. It’s a brilliant drug, but it’s really hard to get into the body. So, I’ve had surgeries to have the port line in place, I’m on my sixth line now in six years, and each line is supposed to last ten to twenty years. It’s because the haem arginate is, it crystallises in the line, so it only lasts about a year. So, I’ve had multiple surgeries, multiple hospital stays. The last surgery I was told will probably be the last line they can fit, because I lost a lot of blood during the operation. So, in terms of the haem arginate, I’m on a time limit essentially […] I’m running out of options now. Which is frightening” (Patient E)

The survey reported that 94% (*n* = 30/32) of patients experienced chronic symptoms between acute attacks. The most common were pain (81%, *n* = 26), fatigue/tiredness (78%, *n* = 25), emotional distress (75%, *n* = 24) and trouble sleeping (56%, *n* = 18). The range of chronic symptoms experienced by patients with AHP were further elucidated in the interviews. Chronic physical symptoms included paralysis, muscle weakness, pain, psychological depression, and difficulty concentrating (Table 9).

**Table 9:** Quotes relating to the types of chronic symptoms experienced

Quote (identifier) by chronic symptom
**Pain**
“The main problem that I experience with the acute attacks, was that I was getting chronic pain in between the acute severe attacks. All day, every day…and I required huge doses of painkillers every single day […] Just to get me out of bed, so that I could get – you know – get dressed and try and live life.” (Patient E)
Psychological depression
“[I experience] psychological depression I suppose, because it completely and utterly ruins your life, well it ruined my life. Whether I say that in another ten years’ time, I might sort of be back on track, but so far I’m still not back on track to where I was five years ago.” (Patient A)
Difficulty concentrating
“I just can’t concentrate on any one thing. It’s just really strange. I almost used to be quite, well, reasonably intelligent but I find […] now sometimes it’s difficult to hold a normal conversation because I can’t think of the words I’m trying to say, [the words] just won’t come and definitely sometimes I feel like I’m stupid because I’m saying the wrong words […] the longest I can tend to concentrate on one thing is maybe about an hour.” (Patient F)
Recovery process/neuropathy/paralysis
“From when the porphyria was [undiagnosed], when I was in hospital and I was paralysed […] I gained sensory-motor neuropathy. I walk with a stick and for long distances I need a wheelchair.” (Patient J)
“Between attacks there is a sort…of a recovery process. So, firstly there is some kind of nerve damage. Then there is a significantly long recovery period, which can be quite painful for [my wife] and reduce her mobility […] [Then my wife] starts doing more and engaging more with the things she likes to do […] and my care responsibilities would go down to very little.” (Caregiver A)

In addition, some participants alluded to a phase separate to or overlapping with the acute attack and chronic symptom phases. Symptoms often did not fully resolve during the patient-labeled ‘recovery process’, with 60% of patients experiencing some form of permanent neurologic damage, even those with AHP who had < 3 attacks per year (Table 9).

Questions relating to the management of chronic AHP symptoms were not included within the survey due to the innate complexity and variability of disease management. The patient and caregiver interviews demonstrated how pain was the predominant chronic symptom experienced, the management of which often involved regular analgesics—including codeine and stronger opioids in more severe cases (Table 10). Furthermore, adequate pain relief was not achievable for every patient due to challenges with treatment tolerability.

4. **Impact of AHP**

**Key finding** Regardless of AAR, AHP heavily impacts the daily life of patients and caregivers. While patients experiencing more frequent attacks generally endured a greater impact on their daily life, patients experiencing less frequent attacks also experienced impacts on all domains.

**Table 10:** Quotes relating to the treatment of chronic symptoms

Quote (identifier)
“I’ve been on morphine now for nearly three and a half years because I have constant pain from attacks.” (Patient C)
“She’d be using a TENS [transcutaneous electrical nerve stimulation] machine and they medicated her with almost ever-increasing doses of morphine, pregabalin, […] tramadol, both quick-release and slow-release. […] She had sublinguals, if it was really bad, she’d have morphine.” (Caregiver B)
“[I] don’t use pain relief during the day because I don’t want to be in that state of being knocked out. Not knocked out but […] I mean not ‘with it’ […] Drowsy in front of my children.” (Patient H)

**Fig. 4:**
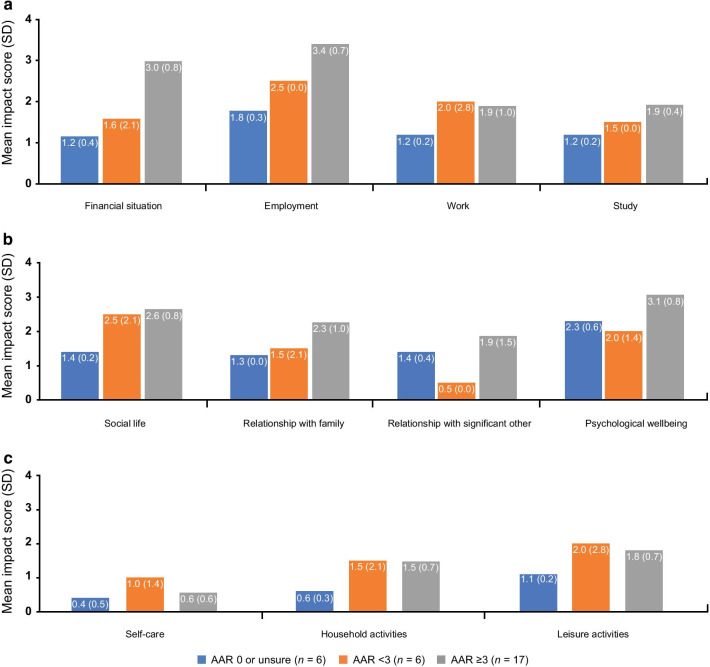
Mean impact scores^a^ by domain in patients with AHP (n = 30). ^a^Mean impact scores based on patient responses were calculated as follows: ‘no impact at all’ = 0, ‘slightly impacted’ = 1, ‘moderately impacted’ = 2, ‘severely impacted’ = 3, and ‘extremely impacted’ = 4. ‘Not applicable’ responses were not included

A total of 30 patients completed the survey question relating to the overall impact on their life. Patients were asked about the extent to which AHP affects aspects of their life on a 5-point scale, ranging from ‘no impact at all = 0’ to ‘extremely = 4’. Mean impact scores were calculated for each domain. Mean results were slightly higher in patients with ≥ 3 attacks per year across most domains, although patients who had < 3 attacks per year clearly experienced an overall impact on various aspects of their life, most notably in ‘employment’, ‘study’, ‘social life’, ‘psychological wellbeing’, and ‘leisure activities’ (Fig. 4).

During the interviews, all patients (*n* = 10) explained how their acute attacks or chronic symptoms affected their work. For acute attacks, this related to whether they had long or frequent hospitalizations or sick leave (Table 11). For chronic symptoms, this included long-term changes in their working ability: for example, needing to move from full-time to part-time, self-employed, or freelance employment (patients, *n* = 2; caregiver, *n* = 1) or to voluntary work or unemployment (patients, *n* = 4); changes in their job role (patients, *n* = 4; caregiver, *n* = 1); or a reduction in the hours able to work (patient, *n* = 1) (Table 11). For some participants, working in shifts and having a demanding job was no longer feasible, while for another, it affected their ability to study.

**Table 11:** Quotes relating to the impact of AHP

Quote (identifier) by area of impact
**Ability to work**
“[For] 18 months I didn’t work at all…I was a sick person at home on benefits.” (Patient A)
“I can’t study when I’m having an attack. I can’t even feed myself, let alone go to the [university] or do the work or read.” (Patient H)
“[I] used to do quite a physical job. [Now] I can’t do retail; I wouldn’t be able to stand for that long really. I wouldn’t be able to do an eight-hour shift…I wouldn’t be able to lift up something heavy at all. 1) Because it hurts and 2) I’m just generally not that strong. So, I do an office job, so I sit behind a computer and take calls and do admin stuff for the council.” (Patient C)
Financial impact
“I completely lost my financial independence.” (Patient E)
“I can’t pay my bills.” (Patient C)
“[I] had to sell the house. Spend my savings.” (Patient G)
Ability to socialize
“It doesn’t do anything for your social opinion of yourself…I am quite proud to be a nurse in social situations and I am a nurse and I work full time and I pay tax. You know I am an upstanding citizen, when you’re on benefits in social situations it can be embarrassing.” (Patient A)
“It does mean that whenever the pain hits, I have to drop everything I do. Cancel all my plans, no matter what they are; cancel weddings, cancel holidays, everything basically…” (Patient E)
“Before porphyria came and reared up…[I] had a normal life. Had a job, had a social life, then porphyria came along, I can’t work anymore, I’m classed as disabled. And I can’t go out and socialise as much as I could do, so it’s changed my life, it’s turned my world upside down.” (Patient J)
Impact on relationships
“More [of] a doctor and a nurse than I am a husband…” (Caregiver B)
“I’ve not had a partner since being ill. But being ill is probably a reason why I’ve not got a partner…You’re this sick person who is on benefits, doesn’t have any money, doesn’t have any prospects, you don’t feel very attractive.” (Patient A)
Ability to carry out personal and household tasks
“If I’ve just come out of hospital and I can’t walk and I’m on crutches or I’m in a wheelchair, then obviously, getting in and out of a bath to have a shower or to just make myself a dinner or make myself a cup of tea isn’t easy because my house isn’t adapted for that. […] eventually when things get worse and I won’t be able to recover, then obviously my house, my flat will have to be adapted.” (Patient C)
“[I] buy food, make dinner, kind of what I describe as the household chores, right. And [wife] might spend an entire day on the sofa. Not because, she doesn’t [want to] help me, but because she’s in too much pain or is just too fatigue[d].” (Caregiver C)
Impact on psychological wellbeing
“I suffer with hallucinations, confusion, I don’t really know what’s going on, I don’t really know where I am, I’m not safe to be alone.” (Patient C)
“I’m only 27 but I feel like I’m 50 something […] sometimes I’ve told my mum before, I don’t even want to be here anymore. But I think like that when I’m in a crisis more. When I’m not, when I’m okay, I feel okay, but it’s just hard to live with. I said to my mum that I think if I didn’t have the children, I don’t think I could, I would live like this, with putting up with the treatment and everything else that [is] involved.” (Patient H)

Financial strain was reported (patients, *n* = 5; caregiver, *n* = 1) as a result of the impact of AHP on the patients’ or caregivers’ ability to work (Table 11). One patient noted the impact of not working on their social esteem and feeling part of society (Table 11).

Social and family life for patients and caregivers was dependent on reducing or changing the habits that existed prior to their AHP diagnosis. Seven patients and two caregivers shared experiences of how they felt their relationship with their partner had changed due to AHP. These changes included their roles as husband or wife and the levels of intimacy within the relationship (Table 11). Another patient, who was single at the time of the interviews, spoke of the difficulties in engaging in any new relationships due to their illness. Impacts on psychological wellbeing, including feeling anxious or depressed, were reported within the survey (18%, *n* = 7). The interviews supported this finding and elaborated further on psychological symptoms of anxiety and depression (Table 11).

The majority of patients reported difficulty in carrying out household tasks such as cooking, shopping, and cleaning (90%, *n* = 9). Mobility (40%, *n* = 4); in personal care such as washing and showering (30%, *n* = 3); and maintaining independence (30%, *n* = 3) were also noted as challenges. The subsequent interviews reinforced that personal independence, the need for care and assistance in carrying out household tasks, and assistance with mobility were dependent on the caregiver and other unpaid support from family (Table 11).

**Fig. 5:**
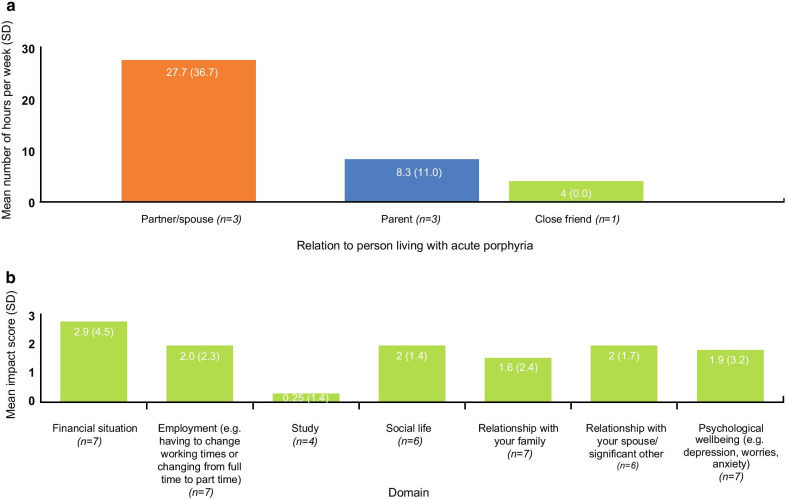
Analysis of caregiver responses. **a** Mean number of hours per week caring for their loved one; **b** Mean impact scores^a^ by domain in caregivers caring for patients with AHP. ^a^Mean impact scores based on patient responses were calculated as follows: ‘no impact at all’ = 0, ‘slightly impacted’ = 1, ‘moderately impacted’ = 2, ‘severely impacted’ = 3, and ‘extremely impacted’ = 4. ‘Not applicable’ responses were not included

The survey captured the average amount of time caregivers spent per week caring for their loved one with AHP (Fig. 5a). Caregivers were also asked to rate how much caring for someone with AHP impacted various aspects of their life, from ‘not at all’ to ‘extremely’. Most caregivers reported financial impact, relationships with spouse, and employment as the most severely impacted domains, while just over half of the caregivers reported a moderate impact on their psychological wellbeing (Fig. 5b). The impact of AHP on caregivers was also captured in the interviews (Table 11).

## Discussion

The objective of this study was to explore the burden of illness of AHP, including the extent to which AHP affects work and finances, social and family life, personal care, independence, and relationships, from both patient and caregiver perspectives. This study included those patients with an AAR of < 3 attacks per year, as well as those with ‘recurrent attacks’ of ≥ 3 per year.

The results of the survey demonstrated that the majority of patients had experienced an acute attack, with a minority of patients never having experienced an acute attack. Of note, 11% (*n* = 4) of patients were unsure if they had ever experienced an attack, highlighting the varied and complex nature of attacks. These patients may have experienced an attack that they perceived to be too ‘mild’ to be considered, or they may have been unsure because of there being no universal definition of an ‘attack’ within AHP. Interestingly, while studies often define severity by the number of attacks, patients and caregivers did not specify that number of attacks was a driver of severity. This may be because they focus on one acute attack at a time, as opposed to a holistic awareness of the number of attacks they have experienced in a year. As seen in Fig. 2, the majority of patients surveyed (60%) also reported that they were able to manage their attacks at home. Attack severity is often attributed to the setting in which the attack is treated; our finding therefore challenges the assumption that if a patient is not presenting in the hospital, they must not be experiencing a severe attack.

The survey reported that 94% of patients experienced chronic symptoms between acute attacks. This supports the research carried out by Gouya et al. [12], which found that most patients experience chronic symptoms as well as acute attacks and that chronic symptoms can also adversely impact daily living and quality of life. This study identified a potential intermediary phase between an acute attack and the experience of chronic symptoms: a ‘recovery phase’ where patients perceive the worst of their attack to be over, while still suffering from the ramifications of the attack (for example, paralysis or muscle weakness). The ‘recovery phase’ can often be so prolonged that patients may progress into a new attack before they have fully recovered. Further research is required to fully understand this phase and the potential cumulative effects on the patient.

Pain was the most frequently reported acute and chronic symptom for AHP patients. Management of pain as a chronic symptom was a key theme throughout the survey and interview responses. While most patients tried to manage their acute attacks at home, difficulty in pain management following an increase in the severity of pain was a key driver for seeking hospital care. Despite this, there was still an overall reluctance to seek medical care as a result of the difficulties patients and caregivers experienced within the hospital setting. As seen in Fig. 3, these difficulties related to a lack of understanding and recognition of AHP among healthcare professionals, leading patients and caregivers to withdraw from the healthcare system and creating a barrier to them seeking treatment. It is therefore important to understand that attack severity and definition may not be best determined by the setting.

The burden of AHP on patients’ and caregivers’ social, family, and working lives is substantial. For both patients and their caregivers, employment, finances, and psychological wellbeing are the aspects of life that were most severely affected by AHP. In this study, disease impact was observed by most patients, regardless of the number of attacks being experienced. However, disease impact did appear to increase for most aspects of quality of life as attack frequency increased, shown in Fig. 4. These are significant findings as most previous studies have only established reduced quality of life in patients experiencing recurrent attacks, with little data being captured for patients experiencing infrequent attacks. Given that AHP typically presents in people between 20 and 45 years old, the lifelong physical, emotional, and economic burden to patients and their families is high, and the subsequent implications for the healthcare system are likely considerable. While a patient’s experience with their disease may ebb and flow, they are nevertheless often left with long-lasting physical, mental, and emotional consequences. There is a clear need to improve education on AHP within the wider healthcare system (e.g., first points of contact for patients), to ensure there is an urgency to treat, to improve patient care, and to prevent further attacks and disease progression.

All patients included in the interviews had experienced at least one attack in the past 2 years, indicating that despite current disease management efforts, attacks could not be fully prevented. It is therefore important to have efficacious and tolerable treatment options available. Where possible, treatment decisions should aim to anticipate cumulative negative effects and minimize social and personal restrictions and the impact of time off work. The availability of additional treatment options for AHP may have a considerable impact on patients, caregivers, and the healthcare system [15].

### Study strengths and limitations

Purposive sampling enabled a lower margin for error and enabled the researchers to target a difficult-to-reach demographic. The majority of participants were females in their 2nd to 4th decades of life at time of diagnosis; while this reflects the gender and age incidence observed for AHP [2, 7], the paucity of experiences from male patients and patients of younger or older ages with AHP is a limitation within this study. Although the number of in-depth interviews conducted was small, results were consistent with those observed in the larger survey, suggesting that the results may be generalized to the population of patients with AHP in the UK. Patients and caregivers also noted that they found the experience cathartic.

Although the patients’ healthcare providers were not required to confirm their diagnosis, patients were asked to select answers reflecting that they had a confirmed diagnosis. These questions included asking what type of porphyria they had and the type of diagnostic testing they had received. Potential researcher bias is a limitation in any patient-reported study methodology, and it should be noted that due to using the snowballing methodology to attain an additional patient and caregiver into the study, this does add to the selection bias of our sample. All interviewed patients had experienced an attack within the past 2 years and had received treatment to manage their attacks; the fact that these were recent attacks may have introduced bias.

Due to the limited number of patients included in this study, subgroup analyses of disease impact scores (Fig. 4) by frequency of attacks could not draw statistical conclusions. Additionally, the number of caregivers within this study was too small to draw any generalizable findings; future work would benefit from looking at a wider caregiver population. This study was designed to establish an understanding of the burden of illness for patients and caregivers, not to explore the effect of time or disease progression on the burden of illness.

## Conclusion

The results of this study provide a snapshot of the burden of AHP and the impact of both acute attacks and chronic symptoms on the lives of a heterogeneous patient population and their caregivers. The qualitative semi-structured interviews provide an in-depth picture of the burden of AHP and support the quantitative results provided by the survey. Further work is needed in the field of AHP to improve the definition of disease burden. It is clear from the results of this analysis that the burden of illness with AHP is high for all patients and caregivers, regardless of the frequency of attacks experienced, and there remain many unmet needs within this population.

## Methods

### Patients

Patient and caregiver data were collected via an online survey of members of the British Porphyria Association (BPA) and/or Porphyria UK, followed by an optional 1-h telephone in-depth interview.

**Fig. 6:**
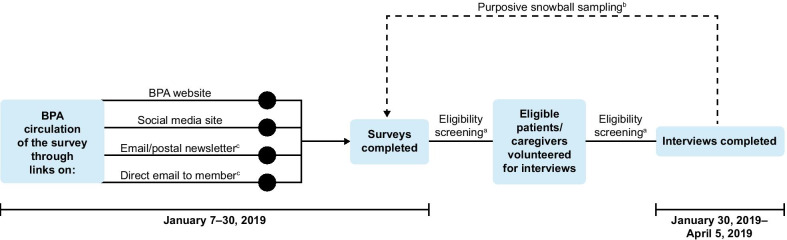
Mixed-method study design. ^a^Table 12 contains the inclusion and exclusion criteria for both the survey and the interviews. ^b^One patient and one primary caregiver were obtained via snowball sampling. ^c^The BPA membership contained 270 members associated with AHP

Purposive sampling was used for both phases using pre-defined selection criteria. Eligibility was confirmed through questions integrated within the survey (Table 12). All patients were included within the survey results regardless of their previous experience of acute manifestations. In addition, snowball sampling via interview participants was used to increase participation in the interviews [16]. All interview participants completed the survey. An overview of the study methodology is provided in Fig. 6. Adverse event reporting was carried out and all personal information was handled in accordance with the General Data Protection Regulation 2018. The study received approval from the Reading Independent Ethics Committee.

**Table 12:** Patient eligibility criteria for the survey and interview phases

**Online survey inclusion/exclusion criteria**
Patients and caregivers were required to be ≥ 18 years old with a confirmed diagnosis (or caring for a patient with a confirmed diagnosis) of AHP, including AIP, VP, HCP and ADP For the purposes of this study a diagnosis of AHP included participant-reported confirmation of elevated ALA testing, elevated PBG testing or positive genetic testing Participants who did not fulfil these criteria were excluded
Interview inclusion criteria
Patients currently treated with prophylactic heme arginate or hormone suppression therapy, or who had experienced at least one acute attack in the past two years that required hospitalization, an urgent healthcare visit or treatment with intravenous heme arginate at home Patients and caregivers were required to give their informed consent for both the survey and the interviews and were considered to have the cognitive ability to adequately understand and answer questions during the interview, in the opinion of the researchers conducting the interview
Interview exclusion criteria
Patients were not interviewed if they were participating in a clinical trial investigating a medicinal product for AHP, or if they had received a liver transplant to treat AHP

### Data analysis

Patient and caregiver responses from the online survey were analyzed using descriptive statistics. Missing data or incomplete responses were analyzed up to the questions that were completed, meaning *n* varies per survey question. Qualitative data from the interviews were thematically analyzed within a pre-defined coding framework, developed and agreed by two independent researchers. Both deductive and inductive coding was used, and newly emerging themes were discussed between the two researchers. Transcripts were coded, applying the coding framework in NVivo^©^.

## Supplementary Information

**Additional file 1: Table S1.** Reported annualized attack rate for patients interviewed.

## Data Availability

All relevant data used in this study have been included in the manuscript. The corresponding author can be contacted if any further information is needed.

## Abbreviations

A&E: Accident & Emergency
AAR: Annualized attack rate
ADP: Aminolevulinic acid dehydratase deficiency porphyria
AHP: Acute hepatic porphyria
AIP: Acute intermittent porphyria
ALA: Aminolevulinic acid
BPA: British Porphyria Association
ED: Emergency department
GnRH: Gonadotropin-releasing hormone
HCP: Hereditary coproporphyria
ICU: Intensive care unit
IV: Intravenous
N/A: Not applicable
PBG: Porphobilinogen
SD: Standard deviation
TENS: Transcutaneous electrical nerve stimulation
VP: Variegate porphyria
