# Supplementary material for: Patient and caregiver experiences of living with acute hepatic porphyria in the UK: a mixed-methods study
Source: Orphanet J Rare Dis. 2021 Apr 26;16:187. doi: 10.1186/s13023-021-01816-2 (PMC8074407; doi:10.1186/s13023-021-01816-2)
Supplement: Supplementary file 1 — Additional file 1: Table S1. Reported annualized attack rate for patients interviewed. [file 13023_2021_1816_MOESM1_ESM.docx]

**Additional material**

**Table S1** Reported annualized attack rate for patients interviewed

| Patients  <3 attacks per year | Patients  ≥3 attacks per year |
| --- | --- |
| Patient A, D, I, J | Patient B, C, E, F, G, H |
